# PCSK9 Induces Rat Smooth Muscle Cell Proliferation and Counteracts the Pleiotropic Effects of Simvastatin

**DOI:** 10.3390/ijms22084114

**Published:** 2021-04-16

**Authors:** Maria Giovanna Lupo, Silvia Marchianò, Maria Pia Adorni, Francesca Zimetti, Massimiliano Ruscica, Maria Francesca Greco, Alberto Corsini, Nicola Ferri

**Affiliations:** 1 Dipartimento di Scienze del Farmaco, Università degli Studi di Padova, 35131 Padova, Italy; mariagiovanna.lupo@unipd.it; 2 Department of Laboratory Medicine and Pathology, University of Washington, Seattle, WA 98195, USA; marchias@uw.edu; 3 Center for Cardiovascular Biology, University of Washington, Seattle, WA 98195, USA; 4 Institute for Stem Cell and Regenerative Medicine, University of Washington, Seattle, WA 98195, USA; 5 Unit of Neurosciences, Department of Medicine and Surgery, Università degli Studi di Parma, 43125 Parma, Italy; mariapia.adorni@unipr.it; 6 Dipartimento di Scienze degli Alimenti e del Farmaco, Università degli Studi di Parma, 43124 Parma, Italy; francesca.zimetti@unipr.it; 7 Dipartimento di Scienze Farmacologiche e Biomolecolari, Università degli Studi di Milano, 20133 Milan, Italy; massimiliano.ruscica@unimi.it (M.R.); mariafrancesca.greco@unimi.it (M.F.G.); alberto.corsini@unimi.it (A.C.); 8 IRCCS, Multimedica, 20099 Milan, Italy

**Keywords:** PCSK9, smooth muscle cells, statins, cholesterol, HMG-CoA reductase

## Abstract

Human atherosclerotic plaque contains smooth muscle cells (SMCs) negative for the contractile phenotype (α-smooth muscle actin) but positive for proprotein convertase subtilisin/kexin type 9 (PCSK9). Thus, we generated rat SMCs which overexpressed human PCSK9 (SMCs^PCSK9^) with the aim of investigating the role of PCSK9 in the phenotype of SMCs. PCSK9 overexpression in SMCs^PCSK9^ led to a significant downregulation of the low-density lipoprotein receptor (Ldlr) as well as transgelin (Sm22α), a marker of the contractile phenotype. The cell proliferation rate of SMCs^PCSK9^ was significantly faster than that of the control SMCs (SMCs^puro^). Interestingly, overexpression of PCSK9 did not impact the migratory capacity of SMCs in response to 10% FCS, as determined by Boyden’s chamber assay. Expression and activity of 3-hydroxy-3-methylglutaryl-coenzyme A reductase (Hmgcr) was significantly increased in the presence of PCSK9, both in SMC^PCSK9^ and after treatment with recombinant PCSK9. The transcriptional activity of sterol regulatory element-binding protein (SREBP) was also increased in the presence of PSCK9, suggesting a direct role of PCSK9 in the control of SRE-responsive genes, like HMGCR. We also observed that cholesterol biosynthesis is elevated in SMC^PCSK9^, potentially explaining the increased proliferation observed in these cells. Finally, concentration-dependent experiments with simvastatin demonstrated that SMCs^PCSK9^ were partially resistant to the antiproliferative and antimigratory effect of this drug. Taken together, these data further support a direct role of PCSK9 in proliferation, migration, and phenotypic changes in SMCs—pivotal features of atherosclerotic plaque development. We also provide new evidence on the role of PCSK9 in the pharmacological response to statins—gold standard lipid-lowering drugs with pleiotropic action.

## 1. Introduction

Clinical studies, together with in vivo and in vitro experiments, established the direct correlation between cholesterol levels, atherosclerosis, and acute coronary syndrome (ACS) [1]. Thus, therapies which aim to reduce low-density lipoprotein cholesterol (LDL-C), such as statins, are currently used for the treatment and prevention of ACS. Statins block cholesterol biosynthesis by inhibiting 3-hydroxy-3-methyl-3-glutaryl coenzyme A (HMG-CoA) reductase, thereby reducing the pool of intracellular lipids [2]. Cells respond to the decreased amount of intracellular cholesterol by activating the sterol regulatory element-binding proteins (SREBPs). These transcription factors undergo proteolytic activation resulting in the induction of LDL receptors (LDLR) and the consequent increased uptake of circulating LDL particles. SREBP activation, however, also leads to upregulation of HMG-CoA reductase and proprotein convertase subtilisin/kexin type 9 (PCSK9). Previous studies indeed showed that statins significantly induce PCSK9, at both mRNA and protein levels [3,4], potentially limiting the hypocholesterolemic and protective action of statins. PCSK9 binds to LDLR and promotes its lysosomal degradation. Thus, upregulation of PCSK9 results in reduced LDL-C uptake by hepatocytes, thereby increasing circulating LDL-C [5].

PCSK9, secreted mainly by the liver, can also be found in neointimal smooth muscle cells (SMCs) of human atherosclerotic plaques [6]. By analyzing SMCs from *Pcsk9^+/+^* and *Pcsk9^−/−^* mice, and by retroviral overexpression, we demonstrated that PCSK9 modulates both SMC proliferation and migration—as well as their switch from a contractile to a synthetic phenotype—in a model of arterial injury [7]. This finding was reinforced by the fact that in human atherosclerotic plaques, SMCs were mainly PCSK9-positive and negative for alpha-actin and were thus considered to be in a synthetic, proliferative phenotype [6].

Available experimental data indicated that neointima formation is not strictly dependent on the presence of hypercholesterolemia, and suggested that the influence of increased cholesterol levels on the arterial response to injury in mice might be dependent on several experimental factors, such as the genotype, the type of vessel involved (carotid versus femoral), the age of the animals, dietary scheme, and some local molecular factors, including PCSK9 [7,8,9,10,11]. This hypothesis was also supported by the evidence that plasma levels correlated with arterial stiffness in humans [12].

The role of PCSK9 in atheroma formation and the effects of statins on PCSK9 expression have been documented in numerous studies [13], however the mechanism behind these remains unclear. The induction of PCSK9 by statins can certainly be ascribed to the activation of the SREBP pathway [3,4], although we previously reported a relevant role of the geranylgeranylated protein Rac1 on the regulation of *PCSK9* transcription, suggesting that isoprenoid regulation might directly contribute to the statin-induced PCSK9 upregulation [14]. Moreover, the isoprenoid pathway also seems to be involved in regulation of cell proliferation. All-*trans* geranylgeraniol (GGOH) is a metabolic derivative in the isoprenoid/cholesterol synthesis pathway and a substrate of geranylgeranyl transferase [15]. Its synthesis is therefore inhibited by statins which, in turn, affect geranylgeranylation or prenylation of proteins involved in a variety of cell functions, including cell proliferation [16]. In particular, two isoprenoids—farnesyl-pyrophosphate (FPP) and geranylgeranyl-pyrophosphate (GGPP)—are substrates of prenyltransferase enzymes involved in the post-translational modification of intracellular proteins [16]. Among many prenylated proteins, Ras, Rho, and Rac1 modulate cellular signaling, differentiation, and proliferation [17], thus potentially explaining the additional pharmacological properties of statins beyond their lipid-lowering action [18].

With the aim to further unravel the role of PCSK9 in atheroma formation and the response to statin treatment, in the present study we generated a new strain of rat SMCs overexpressing human PCSK9 and characterized its phenotype and response to statins.

## 2. Results

### 2.1. PCSK9 Overexpression Significantly Downregulated Ldlr in Rat Smooth Muscle Cells (SMCs)

**Figure 1:**
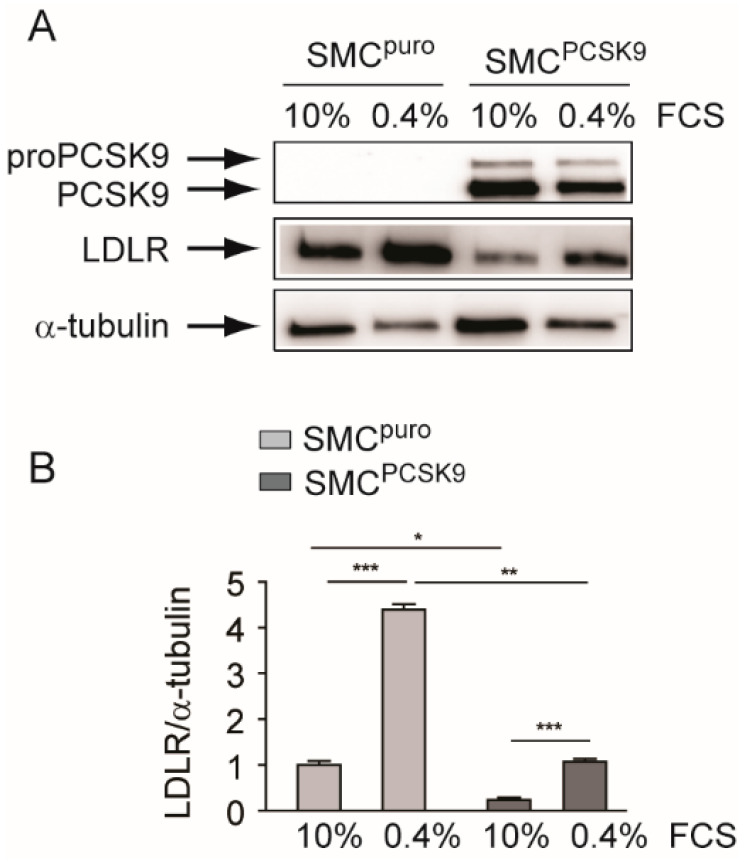
Effect of PCSK9 overexpression on Ldlr in SMCs. SMC^puro^ and SMC^PCSK9^ were seeded in DMEM/10% FCS (3 × 10^5^ cells/well in a 6-well tray). The day after, the medium was changed with DMEM containing either a high (10%) or low (0.4%) concentration of FCS and, after an additional 72 h, the total cell lysates were prepared. (**A**) PCSK9 and Ldlr protein expression were evaluated by Western blot analysis. α-tubulin was used as the loading control. Panel A shows representative images of three independent experiments. (**B**) Densitometric readings for the Ldlr were evaluated using ImageLab software. Ldlr, low-density lipoprotein receptor; PCSK9, proprotein convertase subtilisin/kexin type 9; SMC^PCSK9^, smooth muscle cells overexpressing PCSK9. Differences between conditions were assessed by Student’s *t*-test and one-way ANOVA (when necessary). * *p* < 0.05; ** *p* < 0.01; *** *p* < 0.001.

SMCs from human atherosclerotic plaques showed increased expression of PCSK9 [6], thus we set up an in vitro system that recapitulates the human pathological condition. We isolated rat aorta SMCs and overexpressed human PCSK9 by retroviral infection. We employed an internal ribosomal entry site (IRES)-based retroviral vector expressing the puromycin resistance gene as a selectable second cistron gene, together with PCSK9 as the first cistron gene (SMC^PCSK9^). As a control, the same cell strain was infected with an empty vector expressing only the puromycin resistance gene (SMC^puro^). As shown in Figure 1, PCSK9 was detected by Western blot analysis exclusively in SMC^PCSK9^, while its expression was below the detection limit of the antibody in control SMC^puro^. PCSK9 overexpression strongly downregulated the Ldlr levels in SMCs, in both those cultured with low (0.4%) and high (10%) FCS concentrations (Figure 1A,B). These two conditions differentially regulated the SREBP pathway. Indeed, the incubation of SMCs with 0.4% FCS, i.e., with low lipoprotein concentration, significantly induced the expression of Ldlr, thus suggesting activation of the SREBP pathway and an increase in cholesterol uptake. This effect was observed also in SMC^PCSK9^, although the Ldlr expression was significantly reduced compared with SMC^puro^ (Figure 1A,B).

### 2.2. PCSK9 Overexpression Induced Cell Proliferation in Rat SMCs

**Figure 2:**
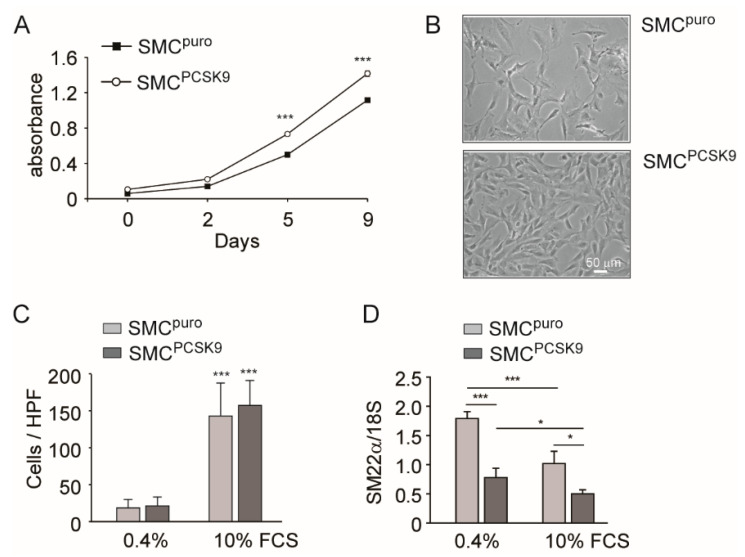
Effect of PCSK9 overexpression on SMC proliferation and migration. (**A**) SMC^puro^ and SMC^PCSK9^ were seeded in DMEM/10% FCS (2 × 10^4^ cells/well in a 24-well tray). Cell number was estimated by Sulforhodamine B staining after 24 h (day 0) and at day 2, 5, and 9. The data are expressed as mean ± SD of absorbance at 570 nm. Error bars (not shown) are within the symbol limits. For each time point, *** *p* < 0.001 SMCs^puro^ vs. SMCs^PCSK9^. (**B**) Representative images of SMC^puro^ and SMC^PCSK9^ after 9 days of growth. (**C**) SMC^puro^ and SMC^PCSK9^ were cultured for 24 h in DMEM containing 0.4% FCS. After 24 h, cells were harvested by trypsinization and migration was measured by Boyden’s chamber chemotactic assay, using FCS as the chemotactic agent at the indicated amount. Transmigrated cells were counted after 6 h of migration in six random high-power fields (HPFs) under high magnification (objective lens 20×). For each cell type, *** *p* < 0.001 10% vs. 0.4% FCS. (**D**) SMC^puro^ and SMC^PCSK9^ were cultured in the presence of low (0.4%) and high (10%) FCS concentrations. After 24 h, total RNA was prepared and the mRNA level of Sm22α was determined by quantitative real-time PCR. * *p* < 0.05 and *** *p* < 0.001. Differences between conditions were assessed by Student’s *t*-test and one-way ANOVA (when necessary).

We previously demonstrated that SMCs freshly isolated from *Pcsk9^−/−^* mice have a slower proliferation rate in response to 10% FCS, and an impaired migratory capacity driven by platelet-derived growth factor BB (PDGF-BB), compared with those isolated from *Pcsk9^+/+^* mice [7]. We then investigated if the overexpression of human PCSK9 in rat SMC^PCSK9^ can directly affect the proliferation and migratory capacity. The proliferation rate of rat SMC^PCSK9^ was faster in response to 10% FCS with a significantly higher cell number at 5 and 9 days postseeding (Figure 2A). This was also confirmed by optical microscopy analysis (Figure 2B), where the monolayer of rat SMCs was more highly compact in SMC^PCSK9^ compared with control. Interestingly, in response to 10% FCS, the migratory capacity of both strains of SMCs was not different, as determined by Boyden’s chamber assay, after 6 h of stimulus (Figure 2C). This suggests that the effects exerted by PCSK9 on cell function depend on the different types of stimuli present in the environment (i.e., growth factor-like PDGF-BB vs. increased lipoprotein in the 10% FCS condition).

We evaluated the expression of phenotypic markers by qPCR reaction under low (0.4%) and high (10%) FCS concentrations. The incubation with 0.4% FCS induced a phenotypic switch from synthetic to contractile phenotype, as evidenced by a significant increase in Sm22α in both SMC^puro^ and SMC^PCSK9^, as compared with the condition with 10% FCS (Figure 2D). However, under both experimental conditions, the overexpression of PCSK9 led to a significant reduction in this contractile marker. These data, together with the increase in proliferation rate, indicate that PCSK9 directly shifts SMCs phenotype to a synthetic one.

### 2.3. PCSK9 Affected HMG-CoA Reductase Expression and Cholesterol Biosynthesis

**Figure 3:**
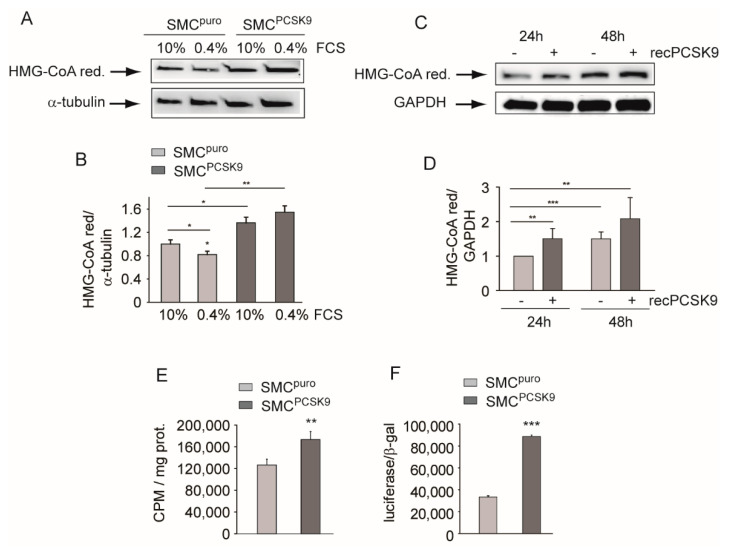
PCSK9 induced Hmgcr and cholesterol biosynthesis. SMC^puro^ and SMC^PCSK9^ were seeded in DMEM/10% FCS (3 × 10^5^ cells/well in a 6-well tray). The day after, the medium was changed with DMEM containing either high (10%) or low (0.4%) FCS concentration and, after an additional 72 h, the total cell lysates were prepared. (**A**) Hmgcr expression was evaluated by Western blot analysis. α-tubulin was used as the loading control. (**B**) Bar graphs of the quantification of Western blot analysis. (**C**) SMCs were cultured in DMEM containing 10% FCS in the presence or absence of 5 µg/mL of human recombinant PCSK9 (recPCSK9) for 24 h and 48 h. At the end of the incubation, the total protein extracts were prepared, and Hmgcr levels were determined by Western blot analysis. GAPDH was used as the loading control. (**D**) Bar graphs of quantification of Western blot analysis. (**E**) SMC^puro^ and SMC^PCSK9^ were cultured in DMEM/10% FCS containing [2-^14^C]-acetate for 48 h. [2-^14^C]-acetate incorporation into cellular sterols was used to assay cholesterol biosynthesis. Each point represents the mean ± SD of triplicate dishes. (**F**) SMC^puro^ and SMC^PCSK9^ were transfected with pGL3-*PCSK9*-D4. Forty-eight hours post-transfection, the medium was replaced with DMEM containing 10% FCS, and, after an additional 24 h, luciferase activities were determined by Neolite reagent. Luciferase activities were normalized to the β-galactosidase activity of the cotransfected pCMV-β construct. Differences between conditions were assessed by Student’s *t*-test and one-way ANOVA (when necessary). * *p* < 0.05; ** *p* < 0.01; *** *p* < 0.001.

Since the mevalonate (MVA) pathway is involved in cell proliferation and differentiation, we determined the expression levels of HMGC reductase (Hmgcr)—the rate limiting step of this pathway—and the cholesterol biosynthesis in SMC^puro^ and SMC^PCSK9^. Western blot analysis showed a significant increase in Hmgcr in SMCs^PCSK9^ compared with control cells, both in the presence of low and high FCS concentrations. As also shown for Ldlr (Figure 1A,B), this difference was particularly evident in cells incubated with low FCS (0.4%) (+1.55-fold) (Figure 3A,B). To further investigate the effect on Hmgcr expression, control SMCs were incubated with a human recombinant PCSK9 protein for 24 h and 48 h (Figure 3C). Hmgcr expression increased over time in the absence of PCSK9, however the incubation of the recombinant protein determined a significant induction of the enzyme (Figure 3C,D).

A significant increase in cholesterol biosynthesis (+36%), determined by measuring the ^14^C-acetate incorporation into cellular cholesterol, was observed in SMCs^PCSK9^ compared with SMCs^puro^ cultured in DMEM/10% FCS (Figure 3E). Since HMG-CoA reductase is under the transcriptional control of SREBP2, we measured the activity of the latter using the luciferase reporter PCSK9 promoter containing sterol regulatory elements (SRE) [19]. After 24 h incubation with medium containing 10% FCS, a strong induction of PCSK9 promoter activity was observed in SMCs^PCSK9^ compared with SMCs^puro^ (Figure 3F). Taken together, these sets of data indicate that PCSK9 determined the activation of the SREBP pathway and the induction of its transcriptionally regulated genes, including the *HMG-CoA reductase*.

### 2.4. PCSK9 Overexpression Partially Counteracted the Inhibition of Cell Migration and Proliferation by Simvastatin

**Figure 4:**
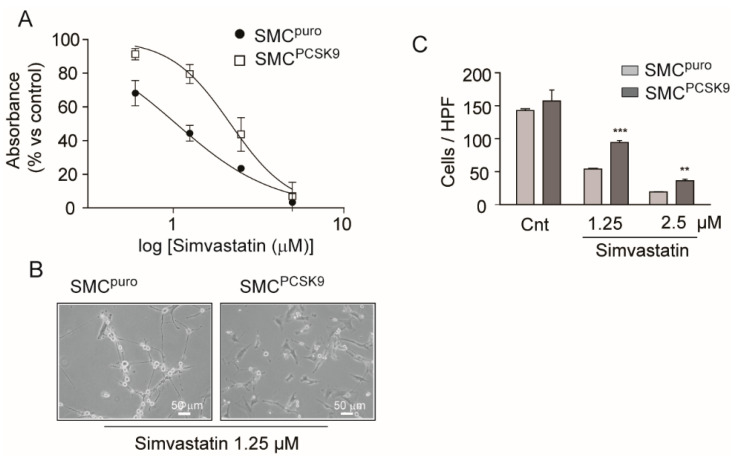
PCSK9-overexpressing cells were partially resistant to simvastatin action. (**A**) SMC^puro^ and SMC^PCSK9^ were seeded in DMEM/10% FCS (2 × 10^4^ cells/well in a 24-well tray). After 24 h, cells were incubated in the presence or absence of the indicated concentration of simvastatin and, in a separate well, the cell number was estimated by Sulforhodamine B (SRB) staining (at time 0). After 72 h of incubation, the cell number was evaluated by SRB and cell proliferation was determined. (**B**) Representative images of SMC^puro^ and SMC^PCSK9^ after 72 h of incubation with 1.5 µM simvastatin in DMEM/10% FCS. (**C**) SMC^puro^ and SMC^PCSK9^ were cultured for 24 h in DMEM containing 0.4% FCS. After 24 h, cells were harvested by trypsinization and incubated in the presence or absence of the indicated concentration of simvastatin during 6 h of migration, measured by Boyden’s chamber chemotactic assay. FCS (10%) was used as the chemotactic agent. Transmigrated cells were counted after migration in six random high-power fields (HPFs) under high magnification (objective lens 20×). Differences between conditions were assessed by Student’s *t*-test. ** *p* < 0.01; *** *p* < 0.001 SMC^PCSK9^ vs. SMC^puro^.

Since statins inhibit SMC proliferation and migration by reducing the intracellular pool of FPP and GGPP, we hypothesized that the increase in the enzymatic mass of Hmgcr and cholesterol biosynthesis may partially alter the pharmacological response to simvastatin of rat SMCs^PCSK9^. We therefore set up a series of experiments aimed at evaluating the inhibitory activity of simvastatin on rat SMCs^puro^ and SMCs^PCSK9^ proliferation and migration. Simvastatin inhibited, in a concentration-dependent manner, the proliferation of both cell types, cultured for 72 h in the presence of 10% FCS (Figure 4A). However, a significantly different potency of the drug was observed between SMCs^puro^ and SMCs^PCSK9^. Indeed, the calculated IC_50_ values were 1.04 ± 1.09 µM and 2.16 ± 1.09 µM for SMCs^puro^ and SMCs^PCSK9^, respectively. This difference was also evident by observing the morphological changes of cells after simvastatin treatment (Figure 4B). Very similar results were observed on the inhibitory effect of simvastatin on rat SMC migration in response to 10% FCS (Figure 4C).

## 3. Discussion

In the present study we provided new evidence on the role of PCSK9 in the biology and pharmacological response to statins of SMC. PCSK9 overexpression in rat SMCs determined a significant downregulation of Ldlr which activated the SREBP transcriptional activity, leading to higher intracellular levels of Hmgcr. Importantly, a strong induction of Hmgcr was also observed in response to the incubation with human recombinant PCSK9. Thus, by using two different experimental approaches, we observed a significant induction of Hmgcr in response to PCSK9. Thus, it is tempting to speculate that the higher amount of HMG-CoA reductase could be the reason for a partial resistance of SMCs^PCSK9^ to the antiproliferative and antimigratory effect of simvastatin.

The positive effect of PCSK9 on rat SMC proliferation of our study is consistent with previous experimental data on mouse SMCs derived from *Pcsk9* knock-out mice [7]. The positive effect of PCSK9 on cell proliferation has also been observed in *PCSK9^−/−^* hepatocytes, after partial hepatectomy, in hepatocyte cell line HepG2, following PCSK9 downregulation by shRNA [20,21] and in human neuroglioma u251 cells [22].

The molecular mechanism by which PCSK9 induces rat SMCs proliferation is still unknown but some hypotheses can be drawn: (1) PCSK9 may change the cellular cholesterol content which plays a key role in membrane fluidity and thus cell proliferation [23]; (2) variation of cholesterol may also alter the formation of membrane lipid rafts—domains involved in critical cell cycle processes such as the control of cell death and survival [23]; (3) PCSK9 could modulate the cholesterol biosynthetic pathway and thus the prenylation process involved in cell proliferation and migration; (4) variation of LDLR or lipoprotein receptor-related protein 1 (LRP1) may also determine a significant change on platelet-derived growth factor receptor (PDGFR) and thus cell proliferation [24].

In line with possible changes to membrane lipid composition, molecular lipidomic analysis of serum from human carriers of a loss-of-function variant in the *PCSK9* gene (R46L) showed a decrease in several cholesteryl esters and short chain fatty acids containing sphingolipid species [25]. However, pharmacological studies conducted in rat SMCs, treated with inhibitors of different enzymatic steps of the MVA pathway, clearly demonstrated that the inhibition of cell proliferation is not due to the inhibition of cholesterol biosynthesis [26]. For instance, the antiproliferative effect of statins occurs when cholesterol synthesis is suppressed by more than 80% [26], suggesting that strong inhibition of MVA production, elicited by statins, might impede the formation of endogenously derived products, such as FPP and GGP, to support cell proliferation. In addition, the antiproliferative effect of statins was observed in cells exposed to a medium containing 10% FCS, which provides an exogenous source of cholesterol. Thus, the most likely explanation for the positive effect of PCSK9 on cell proliferation can be ascribed to the activation of the MVA pathway and prenylation process. However, additional experiments are required to explore this hypothesis.

The most relevant observation of the present study was the fact that SMCs overexpressing PCSK9 showed a partial, and significant, resistance to the antiproliferative and antimigratory action of simvastatin. This is potentially due to the activation of the SREBP pathway, as demonstrated by the analysis of the cholesterol biosynthesis and the *PCSK9* promoter activity, which led to the induction of the HMG-CoA reductase. The increase in the HMG-CoA reductase enzymatic mass—the rate limiting step of the cholesterol biosynthesis pathway—could be the reason for the partial resistance to simvastatin observed in SMCs^PCSK9^. This observation, although obtained in vitro, may have a relevant translational impact considering that many studies confirmed the expression of PCSK9 in medial artery SMCs [6,7,27,28,29,30,31] and that its plasma levels correlated with arterial stiffness [12]. Thus, it is tempting to speculate that patients with higher vascular levels of PCSK9, whose expression has never been correlated with the circulating form of PCSK9, may be less responsive to the direct antiatherosclerotic effect of statins.

Nevertheless, it must be considered that the improvement of the arterial stiffness could be simply related to LDL-C levels, as recently demonstrated in patients with familial hypercholesterolemia treated with high-intensity statins in combination with PCSK9 inhibitors or ezetimibe [32]. However, it is possible that the pleiotropic effects of statins could be more evident after short-term treatment [33].

## 4. Materials and Methods

### 4.1. Reagents

Dulbecco’s Modified Eagle’s medium (DMEM), trypsin-EDTA, penicillin, streptomycin, L-glutamine, nonessential amino acid solution, fetal calf serum (FCS), plates, and Petri dishes were purchased from EuroClone (Milan, Italy). Recombinant human PCSK9 was obtained from Cayman Chemical (Ann Arbor, MI, USA). The recombinant PCSK9 was formulated in 40 mM Tris-HCl, pH 8.0, 110 mM NaCl, 2.2 mM KCl, and 20% glycerol. The endotoxin level of hPCSK9 was 84.8 EU/mL or 47 EU/mg, below the threshold of 0.1 EU/μg requested for cell-based assays [34]. Simvastatin (Merck Sharp & Dohme Research Laboratories, Readington, NJ, USA) was dissolved in 0.1 M NaOH and the pH was adjusted to 7.4. This solution was then sterilized by filtration [14].

### 4.2. Isolation and Generation of SMCs Overexpressing PCSK9

Rat aortic SMCs were isolated from 2-year-old female rats by explant technique as previously described [35] and maintained in DMEM High Glucose supplemented with 10% FCS, 2 mM L-glutamine, and a solution of penicillin/streptomycin (100 U/mL and 100 µM, respectively). The retroviral expression plasmid encoding PCSK9-FLAG tag was constructed using the pBMN-IRES-PURO plasmid [36]. Human PCSK9-FLAG tag cDNA was kindly provided by Professor P. Tarugi (University of Modena, Italy) and subcloned into retroviral expression plasmid by blunt-end ligation. Retroviral infections of rat SMCs at passage 3 were performed as previously described [37], thus generating SMCs^PCSK9^ and SMCs^puro^, the latter being generated through an empty pBMN-IRES-PURO vector as mock cells. SMCs were used between passage 4 and 9.

### 4.3. Western Blot Analysis

The cells were cultured in 6-well trays at a plating cellular density of 3 × 10^5^ cells/well in DMEM/10% FCS. After 24 h, conditioned media were replaced with fresh media with standard or low FCS percentage (10% or 0.4%, respectively), or with specific treatments in DMEM/10% FCS, and incubated for a further 24 h or 48 h as needed. Cells were washed twice with cold PBS and lysed with a solution of 50 mM Tris pH 7.5, 150 mM NaCl, and 1% Nonidet-P40 containing a protease and phosphatase inhibitors cocktail (Merck Life Science S.r.l. Milan, Itay) for 30 min on ice. Protein samples (20 µg) and a molecular mass marker (Bio-Rad, Milan, Italy) were separated using 4–20% SDS-PAGE (Bio-Rad, Milan, Italy) under denaturation and reduction conditions. The protein samples were then dry-transferred to a nitrocellulose membrane using the Trans-Blot Turbo Transfer System (Bio-Rad, Milan, Italy). Nonspecific binding sites were blocked in tris-buffered saline-Tween 20 (TBS-T 20) containing 5% nonfat dried milk for 60 min at room temperature. The blots were incubated overnight at 4 °C with a diluted solution (5% nonfat dried milk) of the following primary antibodies: anti-PCSK9 (rabbit polyclonal antibody, GeneTex GTX129859; dilution 1:1000), anti-LDLR (rabbit polyclonal antibody, GeneTex GTX132860; dilution 1:1000), anti-HMG-CoA reductase (rabbit polyclonal antibody, GeneTex GTX54088; dilution 1:400), anti-α-tubulin (mouse monoclonal antibody, clone DM1A, Sigma T6199; dilution 1:2000), and anti-GAPDH (rabbit polyclonal antibody, GeneTex GTX100118; dilution 1:3000). The membranes were washed three times with TBS-T and exposed for 90 min at room temperature to a diluted solution (5% nonfat dried milk) of the secondary antibodies (peroxidase-conjugate goat antirabbit and antimouse, Jackson ImmunoResearch; dilution 1:5000, cod. 111-036-045 and 115-036-062, respectively). Immunoreactive bands were detected by exposing the membranes to Clarity Western Enhanced ChemiLuminescence (ECL) chemiluminescent substrates (Bio-Rad, Milan, Italy) for 5 min, and images were acquired with the Azure c400 Imaging System (Aurogene, Rome, Italy). The densitometric readings were evaluated using ImageLab software (Bio-Rad, Milan, Italy).

### 4.4. Reverse Transcription and Quantitative PCR (RT-qPCR)

The cells were cultured in 48-well plates at a plating cellular density of 8 × 10^4^ cells/well in DMEM/10% FCS. After 24 h, conditioned media were replaced with fresh media with standard or low FCS percentage (10% or 0.4%, respectively), and incubated for a further 24 h. Cells were then washed twice with cold PBS and total RNA was extracted using the iScript^TM^ RT-qPCR Sample Prep reagent (Bio-Rad, Milan, Italy), according to the manufacturer’s instructions [38]. TranScriba 1step PCR Mix SYBR kit (A&A Biotechnology, Gdansk, Poland) was used for qPCR, along with specific primers for the selected genes (*Sm22α* forward 5′-ATCCTATGGCATGAGCCGTG-3′; *Sm22α* reverse 5′-CAGGCTGTTCACCAACT TGC-3′; *18S* forward 5′-CGGCTACCACATCCACGGAA-3′; *18S* reverse 5′-CCTGAATTGTTATTTTTCGTCACTACC-3′). The analyses were performed with the CFX96 Touch Real-Time PCR Detection System (Bio-Rad, Milan, Italy) with cycling conditions of 50 °C for 10 min, 95 °C for 1 min, and a repetition of 40 cycles at 95 °C for 15 s followed by 30 s at 60 °C. The data were expressed as Ct values and used for relative quantification of targets with ΔΔCt calculations. The ΔΔCt values were determined by multiplying the ratio value between the efficiency of specific primers and housekeeping 18S. The efficiency was calculated as ((10ˆ(−1/slope)) − 1) × 100.

### 4.5. Cholesterol Biosynthesis Assay

The cells were seeded in 12-well plates. After 24 h, the cells were incubated in DMEM containing 10% FCS in the presence of [2-^14^C] acetate (2 µCi/mL) for 48 h. The synthesis of cholesterol was then determined by measuring the incorporation of radioactive acetate into total cellular sterols [39]. Cell monolayers were washed with phosphate-buffered saline (PBS) and digested with 0.1 M NaOH overnight. Aliquots were saponified at 60 °C for 1 h in alcoholic KOH after the addition of 1,2-^3^H-cholesterol as an internal standard (10^5^ cpm/sample). The unsaponifiable material was extracted with low-boiling point petrol ether and counted for radioactivity. To evaluate the incorporation of labeled acetate into cellular sterols, these were separated from the unsaponifiable fraction via thin-layer chromatography using petroleum ether (boiling point: 40–60 °C)/diethyl ether/acetic acid (70:30:1). Radioactivity was measured by liquid scintillation counting. The data were expressed as counts per minute of [2-^14^C] acetate incorporation into total cellular sterols per milligram of protein.

### 4.6. Luciferase Reported Promoter Activities Assay

SMC^puro^ and SMC^PCSK9^ were transfected with the plasmid pGL3-PCSK9-D4 containing the 5′ flanking region of the human *PCSK9* gene from −440 to −94, relative to the ATG start codon in front of the luciferase coding sequence. To measure the human *PCSK9* promoter activity, SMCs were seeded in 48-well plates at a density of 4 × 10^5^ cells per well. The day after, cells were transiently transfected with pGL3-PCSK9-D4 with TurboFect transfection reagent (Thermo Fisher, Milan, Italy). Forty-eight hours post transfection, cells were incubated with DMEM/0.4% FCS for an additional 24 h. Luciferase activities were measured using Neolite reagent (Perkin Elmer, Milan, Italy) according to the manufacturer’s instructions [40].

### 4.7. Proliferation Assay

Cell proliferation was evaluated through sulforhodamine B (SRB) assay as an indirect estimation of cell number, as previously described [41]. In brief, 8000 cells/well (both SMC^puro^ and SMC^PCSK9^) were seeded in a 96-well tray in DMEM/10% FCS and the SRB assay was performed at different time points, in quadruplicate, for three different experiments. For the determination of the antiproliferative effect of simvastatin, SMC^puro^ and SMC^PCSK9^ were seeded at a density of 8000 cells/well. The day after, cells were incubated in DMEM/10% FCS in the presence or absence of increasing concentrations of simvastatin. After 72 h the SRB assay was performed. At time zero, just before the addition of simvastatin, four wells were used for cell number determination by SRB analysis. These absorbance values were subtracted from those obtained after 72 h.

### 4.8. Cell Migration Assay

The cell migration assay was performed using a Boyden’s chamber. The polycarbonate membrane (Biomap, Milan, Italy) was coated with gelatin solution (1% in physiologic solution). Cell migration was induced with FCS at 0.4% and 10%, accordingly. Migrated SMCs were stained with the Diff-Quik staining set (Biomap, Milan, Italy) and counted using the ImageJ software from six random high-power fields (HPFs, objective lens 20×).

### 4.9. Analysis of the Data

Statistical analysis was performed using the Prism statistical analysis package version 6.0 (GraphPad Software, San Diego, CA, USA). Data are given as the mean ± SD of three independent experiments. To compare differences between two conditions, *p* values were determined by Student’s *t*-test. Otherwise, differences between treatment groups were evaluated by one-way ANOVA. A probability value of *p* < 0.05 was considered statistically significant.

## 5. Conclusions

Our study further supports a direct role of PCSK9 in vascular biology and SMC migration and proliferation—two pivotal features of atherosclerosis—and adds a new pharmacological aspect in the influence of PCSK9 on the pleiotropic effects of statins. Further pharmacological studies with statins in an in vivo model of neointimal hyperplasia are required to confirm our observation.

## Data Availability

The data presented in this study are available on request from the corresponding author.
